# Effect of Sucrose Ingestion at the End of a Critical Window that Increases Hypertension Susceptibility on Peripheral Mechanisms Regulating Blood Pressure in Rats. Role of Sirtuins 1 and 3

**DOI:** 10.3390/nu11020309

**Published:** 2019-02-01

**Authors:** Vicente Castrejón-Téllez, Mariana Villegas-Romero, Israel Pérez-Torres, Gabriela Zarco, María Esther Rubio-Ruiz, Elizabeth Carreón-Torres, Eulises Díaz-Díaz, Oscar Emanuel Grimaldo, Verónica Guarner-Lans

**Affiliations:** 1 Department of Physiology, Instituto Nacional de Cardiología “Ignacio Chávez”, Juan Badiano 1, Sección XVI, Tlalpan, Mexico City 14080, Mexico; vcastrejn@yahoo.com.mx (V.C.T.); mvromero21@gmail.com (M.V.-R.); esther_rubio_ruiz@yahoo.com (M.E.R.-R.); 11630@udec.edu.mx (O.E.G); 2 Department of Pathology, Instituto Nacional de Cardiología “Ignacio Chávez”, Juan Badiano 1, Sección XVI, Tlalpan, Mexico City 14080, Mexico; pertorisr@yahoo.com.mx; 3 Department of Pharmacology, Instituto Nacional de Cardiología “Ignacio Chávez”, Juan Badiano 1, Sección XVI, Tlalpan, Mexico City 14080, Mexico; gabriela0304@hotmail.com; 4 Department of Molecular Biology, Instituto Nacional de Cardiología “Ignacio Chávez”, Juan Badiano 1, Sección XVI, Tlalpan, Mexico City 14080, Mexico; qfbelizabethcm@yahoo.es; 5 Department of Reproductive Biology, Instituto Nacional de Ciencias Médicas y Nutrición “Salvador Zubirán”, Vasco de Quiroga 15, Sección XVI, Tlalpan, Mexico City 14000, Mexico; eulisesd@yahoo.com

**Keywords:** hypertension, sucrose ingestion, endothelial nitric oxide synthase, oxidative stress, fatty acids, critical window

## Abstract

Susceptibility to develop hypertension may be established during early stages of life that include the intrauterine period, infancy and childhood. We recently showed that blood pressure increased when rats reached adulthood when sucrose was ingested for a short-term critical window from postnatal day 12 to 28 in the rat, which corresponds to days around weaning. Here, we studied several factors that might participate in the increased susceptibility to hypertension when adulthood is reached by analyzing the changes produced at the end of the sucrose ingestion during this critical period. Body weight of the rats at the end of the sucrose period was decreased even if there was an increased ingestion in Kcal. We found an increase in blood pressure accompanied by a decrease in endothelial nitric oxide synthase (eNOS) expression in the aorta. When insulin was administered to rats receiving sucrose, glucose in plasma diminished later than in controls and this slight insulin resistance may reduce nitric oxide synthase action. Oleic acid that modulates eNOS expression was increased, lipoperoxidation was elevated and total non-enzymatic anti-oxidant capacity was decreased. There was also a decrease in SOD2 expression. We also studied the expression of Sirt1, which regulates eNOS expression and Sirt3, which regulates SOD2 expression as possible epigenetic targets of enzyme expression involved in the long- term programming of hypertension. Sirt3 was decreased but we did not find an alteration in Sirt1 expression. We conclude that these changes may underpin the epigenetic programming of increased susceptibility to develop hypertension in the adults when there was exposure to high sucrose levels near weaning in rats.

## 1. Introduction

Increased propensity to develop hypertension may be determined during early stages of life that include the intrauterine period, infancy and childhood [1]. Perturbations during gestation and lactation that induce low birth weight of offspring have been studied in fetal programming of hypertension [2,3,4,5]. Administration of high sucrose during pregnancy and before it in addition to its administration during lactation and the first days after weanling elevated susceptibility to develop hypertension when adulthood was reached [6]. A diet containing high salt before and during pregnancy, lactation and the early weaning period also elevated the susceptibility to high blood pressure when rats reached the adult age [7]. A high salt diet only during gestation, increased incidence in female but not in male offspring to develop hypertension when adults [8]. 

We recently showed that blood pressure increased when sucrose was ingested for a short-term critical window from postnatal day 12 to 28 in the rat which corresponds to days around weaning [9]. The increase found was similar to hypertension induced in rats with metabolic syndrome that received sucrose for 7 months. In rats that received sucrose for the brief lapse of time, the expression of endothelial nitric oxide synthase (eNOS) and superoxide dismutase 2 (SOD2), and total antioxidant capacity were decreased as much as in metabolic syndrome rats. We concluded that high sugar diets during this possible critical window increase hypertension predisposition in adulthood. In the present paper, we analyze the alterations in three possible mechanisms underlying expression of eNOS and consequently hypertension at the moment when the critical window ends (postnatal day 28) [9]. The mechanisms studied were: (a) through the effect of insulin on vasoreactivity that involves eNOS through the phosphatidyl inositol 3 phosphate (PI3K)/ protein kinase B (PKB or Akt) and mitogen activated protein kinase (MAPK) pathway [10], (b) through an elevation of free fatty acids, specifically oleic acid, that decreases eNOS activity [9,11] and c) through oxidative stress (OS) since reactive oxygen species (ROS) are generated by uncoupled eNOS and other enzyme. ROS react with NO decreasing its bioavailability favoring vasoconstriction [12].

Alterations in these peripheral pathways may be epigenetically programmed. Epigenetic marks might be elicited by high glucose and insulin, causing elevated susceptibility to hypertension. Mitochondrial metabolism leads to the production of molecules that form part of transcription factors and determine the activity of enzymes involved in the establishment or deletion of epigenetic marks [13]. Alterations in the production of these metabolites caused by diet changes might determine the synthesis and liberation of vasoconstrictor and vasodilator substance in the vessels modify the lipidic profile or alter OS that finally regulate tension development. The development of hypertension in the adult might be favored by these changes [14]. Programming during the critical window could be due to the epigenetic effects of silent information regulator T (sirtuins; Sirt) protein expression. Sirts are a family of 7 different NAD^+^ dependent histone deacetylases and ADP-ribosiltransferases that depend on the diet and that regulate the expression of some genes playing important roles in the regulation of vasoreactivity [15]. AMP- activated protein kinase (AMPK) phosphorylation is elevated by Sirt1, and this regulator also reduces the oxidative damage biomarkers [16]. Sirtuin 3 (Sirt3) is related to inflammatory responses that regulate the expression of inducible nitric oxide synthase (iNOS). Sirtuin 3 has also been described to epigenetically regulate SOD expression [17]. Therefore, in this paper, we also explored if Sirt1 and Sirt 3 expressions were modified at the end of the critical window and might participate in the programming of adult hypertension by regulating eNOS and SOD expression.

## 2. Materials and Methods

### 2.1. Animals, Experimental Groups and General Characteristics of the Experimental Groups

Experiments in animals were approved by the Laboratory Animal Care Committee of the Instituto Nacional de Cardiología “Ignacio Chávez” (Mexico) and were conducted in agreement with the animal research ethical guidelines from our Institution (acceptance of protocol by the committee on the 26/02/2013).

Male newborn rats, with a well-known birth date, were assigned as a litter of 8 pups to each mother. Experimental sucrose period rats (SP), were given 30% sucrose in drinking water (the mother with the 8-pup litter) from day 12 to 21 when weaning occurred, and administration of sucrose continued only to the pups from day 21 to day 28. Control rats received tap water during these days. Purina 5001 rat chow (Richmond, IN) *ad libitum* was available during the whole experimental period. The animals were kept under controlled temperature and a 12:12-h light-dark cycle. At least 6 rats belonging to 3 different litters from each group were used. 

During postnatal days 25 to 28 a group of control and SP rats were placed in metabolic cages to determine water and food intake as well as total kilocalories (Kcal) ingested. Body weight was determined on day 28.

### 2.2. Blood Pressure and Biochemical Determinations

For mean arterial blood pressure (BP), six 28-day old rats from 3 litters of control and SP cages, that had undergone overnight fasting for 12 h were weighed and anesthetized via an intraperitoneal injection of 50 mg/Kg of sodium pentobarbital (Anestesal; Pfizer, Mexico) to reach a state of surgical anesthesia. An intra- tracheal tube was placed to allow proper respiration. A catheter filled with Hartmann solution:heparin (3:1) was inserted in the left cranial carotid artery and connected to a blood pressure transducer that sent the signal to a previously calibrated polygraph VR-6 simultrance recorder (Model M4-A, Electronics for Medicine/Honeywell, White Plains, NY, USA) connected to a specifically designed system that transforms the analog signal from this apparatus to a digital one. Five min of recuperation after surgery were allowed before the register was performed. The mean of five independent determinations was calculated. After blood pressure determination, the animals were sacrificed.

For the remaining biochemical determinations, six to eight SP and control rats from three different litters that had undergone overnight fasting (12 h) were killed by decapitation and blood was collected. The aortas and abdominal fat were dissected. Abdominal fat was weighed. The thoracic aortas were dissected and cleaned from surrounding tissue. Blood was spun and serum was separated by centrifugation at 600 g during 15 min at room temperature. Tissues and serum were stored at −70 °C until needed. Rats used for biochemical determinations and tissue obtainment were different from those used for blood pressure determination

A commercial radioimmunoassay (RIA) specific for rat (Linco Research, Inc., St. Charles, MO, USA) was used to determine serum insulin. The assay had a sensitivity of 0.1 ng/mL and 5 and 10% intra- and inter-assay coefficients of variation. An enzymatic SERA-PAK^®^ Plus assay from Bayer Corporation (Bayer Corporation, Sées, France) was used to determine glucose concentration. The homeostasis model assessment of insulin resistance (HOMA-IR) was used as the physiological index of insulin resistance. The HOMA-IR was calculated from the fasting glucose and insulin concentrations by the following formula [18]:

(Insulin (µU/mL) × glucose (in mmol/L)/22.5)(1)

Insulin tolerance index was performed by injecting insulin intraabdominally (1U insulin/kg diluted in 100 µL saline solution) in six fasting control and SP rats from 3 different litters and then taking blood samples from the tail at 15, 30, 60, 90 and 120 min in conscious animals. Glucose was measured with a blood glucose meter (Abbot, Free Style Optium, UK) using standard reactive glucose strips. The rats used were different from those employed for blood pressure determination and biochemical determinations.

Total cholesterol (TC), plasma triglyceride, fatty acids (FA) and non-esterified fatty acids were determined by a previously described method [9,19,20].

### 2.3. Thoracic Aorta Homogenization. 

Homogenization of pools from 3 thoracic aortas from different rat pups was done using a lysis buffer (25 mM HEPES, pH = 7.5; 100 mM NaCl, 10% Glycerol, 1% Triton-X100, 7 mg/mL sodium deoxycholate) supplemented with a mixture of protease inhibitors (1 mM PMSF, 10 µg/mL pepstatin A, 10 µg/mL leupeptin and 10 µg/mL aprotinin) (Sigma Chemical Co., St. Louis, MS, USA) as previously described [9]. The Bradford method was employed to determine protein concentration (Protein assay, Bio-Rad laboratories) [21].

### 2.4. Lipoperoxidation (LPO) and Total Antioxidant Capacity.

A standard method was used to determine LPO, a marker of damage by free radicals [22]. The calibration curve was obtained using tetraethoxypropane as standard [22].

The total antioxidant capacity was determined in 100 µg of protein from the thoracic aorta homogenate by a previously described method [9]. The calibration curve was obtained using Trolox [22].

### 2.5. Endothelial Nitric Oxide Synthase, SOD1 and 2 and Sirt1 and 3 Immunoblotting 

For eNOS, Sirt1 and Sirt3, SOD 1 and SOD2 detection, 50 μg of total proteins were separated on SDS-PAGE and electrophoretically blotted onto polyvinylidenefluoride membranes, as previously reported [9]. Membranes were incubated overnight with a 1:1000 dilution, at 4 °C with primary antibodies, rabbit anti-eNOS (sc-376751), mouse anti-SOD1 (sc-271014) and goat anti-SOD2 (sc-18503) rabbit anti-Sirt1 (sc-15404) (all from Santa Cruz Biotechnology, Santa Cruz, CA, USA) and goat anti-Sirt3 (ab118334) (from Abcam, Cambridge, MA, USA). Then the membranes were rinsed three times with TBS-T buffer and incubated 3 h at room temperature with horseradish peroxidase conjugated secondary antibodies, dilution 1:10,000 (Santa Cruz Biotechnology). All blots were incubated as a control with GAPDH antibody (sc-365062) (Santa Cruz Biotechnology, Santa Cruz, CA, USA). A chemiluminescence assay (Clarity Western ECL Substrate, Bio-Rad Laboratories, Inc., Hercules, CA, USA) was used to detect the protein. Chemiluminescence was evaluated in X-ray films (AGFA, Ortho CP-GU, Agfa HealthCare NV, Mortsel, Belgium). A GS-800 densitometer (including Quantity One software from Bio-Rad Laboratories, Inc.) was used to obtain images from each film. The values of each band density are expressed as arbitrary units (AU).

### 2.6. Statistical Analysis

Results are expressed as mean ± standard errors of the mean (SEM) from 4–8 different animals coming from at least 2 litters or from 4 to 6 determinations from different aortic artery homogenates (from pools of 3 aortas) from different rats and different litters. Comparisons between two values was done by Student’s t test or when more values were tested, analysis of variance (ANOVA) or ANOVA on ranks followed by Student-Newman-Keuls or Dunn´s tests were used, depending on whether the data were normally distributed or not. The Sigma Stat program (Systat Software Inc., San Jost, CA, USA) was used. Differences were considered statistically significant when *p* < 0.05.

## 3. Results

### 3.1. Body Variables, Food Intake, Blood Pressure and eNOS Expression 

Body weight was significantly diminished in 28-day old rats that received sucrose from postnatal day 12. Visceral adipose tissue was not modified. When rats were placed in metabolic cages it was observed that water and food intake were decreased by the addition of sucrose but total Kcal obtained from the diet plus the sucrose in the drinking water were increased (Table 1). 

**Figure 1:**
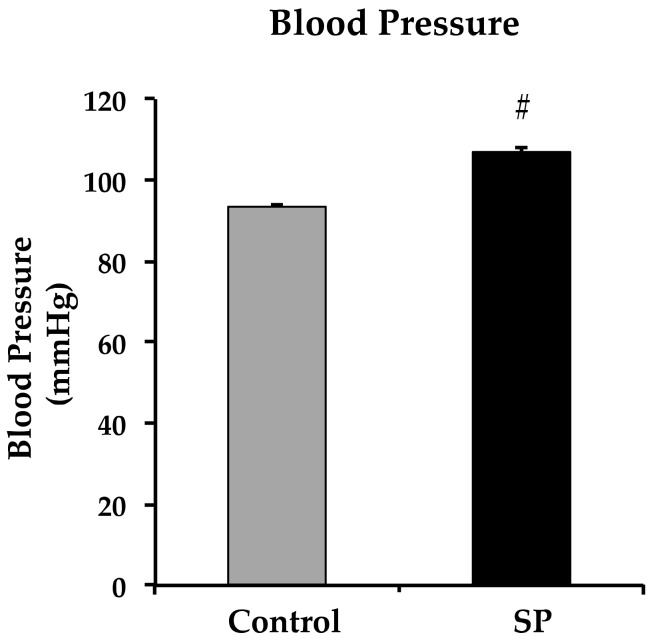
Changes in blood pressure in control and sucrose period rats. # *p* ≤ 0.05. *n* = 6, from 2 different litters. Data represent mean ± SEM, # *p* < 0.05.

Blood pressure was significantly increased in 28-day old rats that received sucrose (Figure 1). 

**Figure 2:**
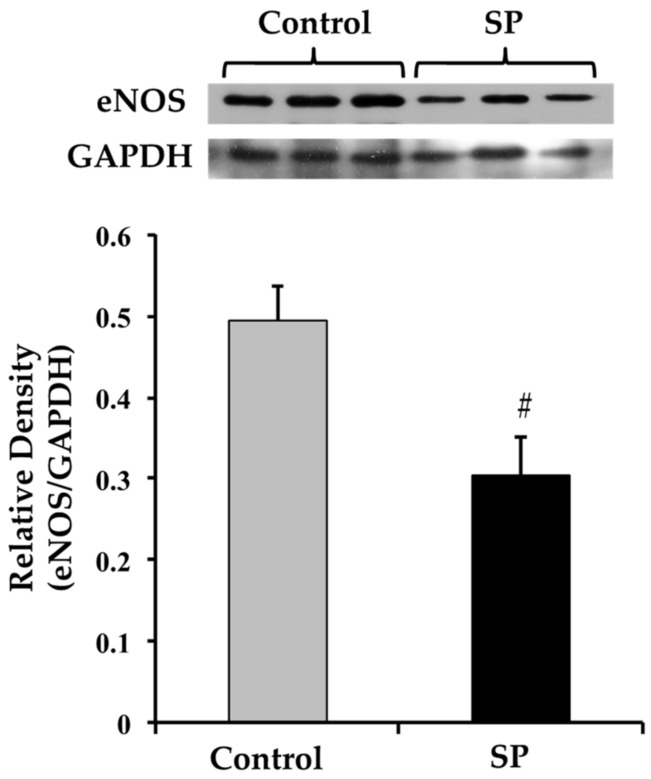
The eNOS expression in aortas from control and sucrose period rats (SP). Data represent mean ± SEM, # *p* < 0.05, *n* = 4 (4 different homogenates from pools of 3 aortas from different rat pups and litters). A representative Western blot analysis is shown above the graph.

The eNOS expression was significantly decreased in rats receiving sucrose (Figure 2).

### 3.2. Changes in Insulin, Glucose and Insulin Resistance that Could Underlie Alterations in Blood Pressure

There were no significant changes in glucose or insulin in plasma. Therefore, HOMA-IR index was not modified (Table 2). 

**Figure 3:**
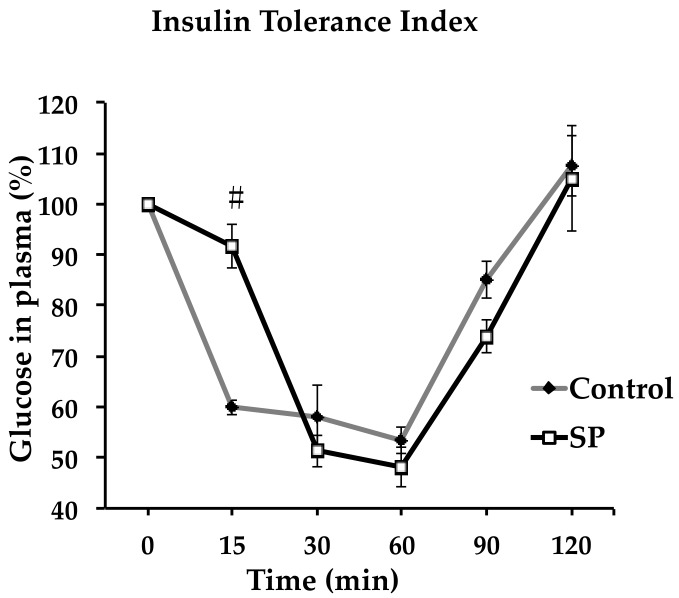
Insulin tolerance index in control and sucrose period rats. Measurements were taken at different times: 15, 30, 60, 90 and 120 min. 100% glucose in plasma corresponds to the value in mg/dl reported in table 2. SP, sucrose period. Data represent mean ± SEM, # *p* < 0.05, *n* = 6.

When the insulin tolerance index was performed, glucose level decreased later in rats that had received sucrose (Figure 3). There was a significant difference (*p* < 0.05) at 15 min after the insulin administration.

### 3.3. Changes in the Lipidic Profile that Could Underlie Changes in Vasoreactivity in Sucrose Period Rats

Table 3 shows that rats receiving sucrose developed hypertriglyceridemia and HDL-cholesterol was diminished. There were no significant changes in total cholesterol and non-HDL cholesterol. 

**Figure 4:**
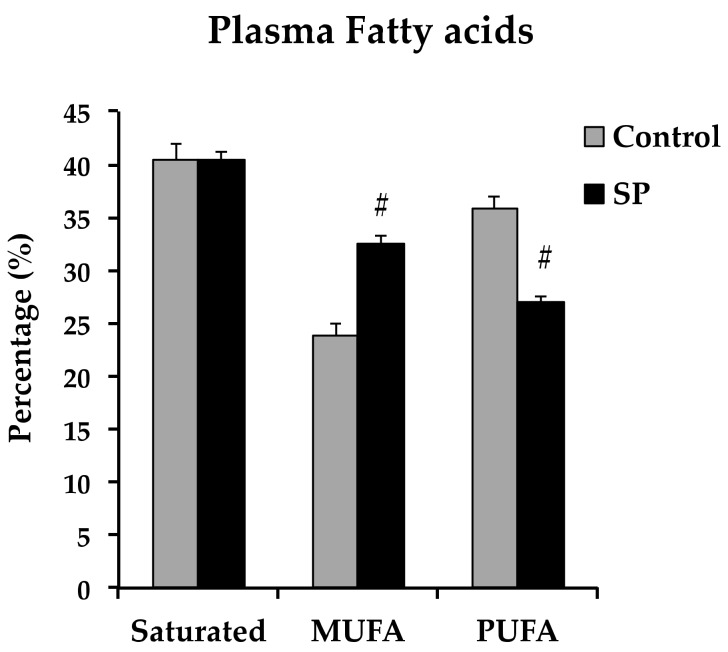
Plasma saturated fatty acids, monounsaturated (MUFA) and polyunsaturated fatty acids (PUFA), in control and sucrose period rats (SP). Data represent mean ± SEM, # *p* < 0.05, *n* = 6.

Saturated fatty acids remained unchanged, but monounsaturated fatty acids were increased while polyunsaturated fatty acids were decreased in the SP group (Figure 4). 

Regarding individual fatty acids, oleic acid was significantly increased, while stearic and lionoleic acids were decreased (Table 4).

### 3.4. Oxidative Stress that Could Underlie Blood Pressure Alterations in the Sucrose Period Rats

**Figure 5:**
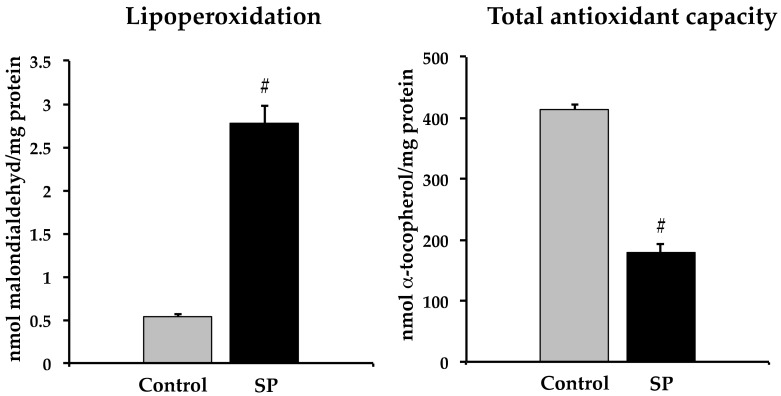
Lipoperoxidation and total antioxidant capacity in the homogenized tissue from the thoracic aorta from control and sucrose period rats. Data represent mean ± SEM, # *p* < 0.05, *n* = 6 (6 different homogenates from pools of 3 aortas from different rat pups and litters).

Lipoperoxidation was significantly increased in rats that received sucrose, while the total antioxidant capacity was decreased (Figure 5). 

**Figure 6:**
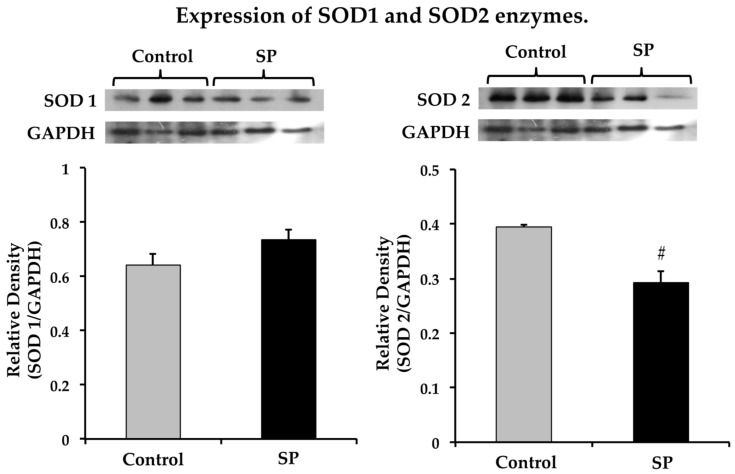
Superoxide dismutase 1 and 2 expression in the homogenized tissue from the thoracic aorta from control and sucrose period (SP) rats. Data represent mean ± SEM, # *p* < 0.05, *n* = 4 (4 different homogenates from pools of 3 aortas from different rat pups and litters). A representative Western blot analysis is shown above the graphs.

In addition to analyzing the non- enzymatic antioxidant capacity, we explored changes in some antioxidant enzymes. SOD1 expression tended to increase but the change was not statistically significant and there was a decrease in SOD2 expression (Figure 6).

### 3.5. Sirtuin 1 and Sirtuin 3 Expression

**Figure 7:**
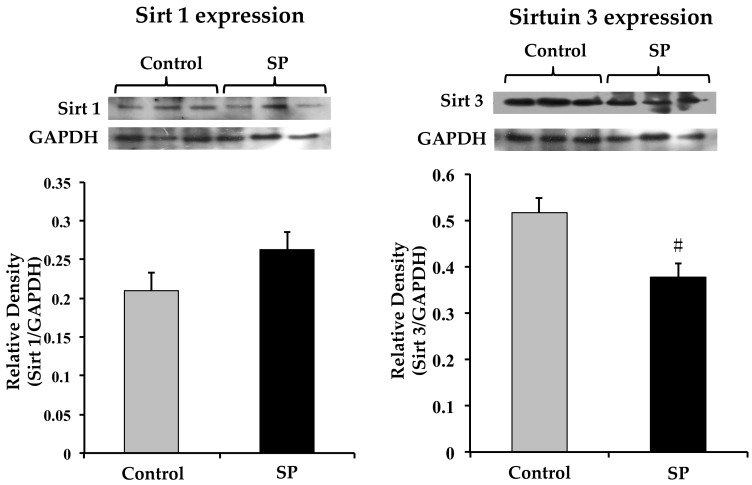
Sirt 1 and Sirt 3 expression in the homogenized tissue from the thoracic aorta from control and sucrose period rats. Data represent mean ± SEM, # *p* < 0.05, *n* = 4 (4 different homogenates from pools of 3 aortas from different rat pups and litters). A representative Western blot analysis is shown above the graphs.

Although a tendency to an elevated expression of Sirt1 that regulates eNOS expression in rats receiving sucrose was observed, the change was not statistically significant (Figure 7). There was a significant decrease in Sirt3 expression that regulates SOD2 protein expression (Figure 7).

## 4. Discussion

At the end of the critical window, rats that received sucrose had increased systolic blood pressure values. We found a decrease in eNOS expression in the aorta. Glucose levels decreased later after insulin administration; thus, a slight insulin resistance which may decrease eNOS activity. Elevated oleic acid levels that modulate eNOS expression were found, lipoperoxidation was elevated and total non-enzymatic anti-oxidant capacity was decreased. There was also a decrease in SOD2 expression. Sirt3 which regulates SOD2 expression was decreased but we did not find an alteration in Sirt1 expression. These changes might participate in the epigenetic mechanisms that cause an increase in blood pressure when the rats reach adulthood. The study of this critical window having effects on hypertension is novel and the possible implications of changes in nutrition impacting on the development of hypertension during adulthood might help prevent the high risk of this disease in adult individuals from early stages of development. In a previous paper [9] we described that blood pressure is increased in adult rats that received sucrose during the same lapse of time as the one used in this paper and were then allowed to drink tap water during 6 months. The increase in blood pressure in these rats was of a similar magnitude to that found after a long- term exposure to sucrose (for 7 months). Rats that received sucrose for 7 months also developed metabolic syndrome. 

Sucrose ingestion by the mother has effects on the composition of the breast milk suckled by the pups. Milk composition is influenced by the dietary protein/carbohydrate ratio which regulates the expression of metabolic genes in the mother [23,24,25,26,27]. Sucrose also acts directly on the newborns since during the pre- weaning period and after weaning the rat pups drink the water that is provided in the cage. To better characterize sucrose ingestion, we placed 28-day-old control and SP pups in metabolic cages. Water and food consumption by the SP pups was diminished, but the pups consumed more daily Kcal equivalents. 

### 4.1. Changes in Body Weight and Food Consumption

The decrease in body weight in sucrose fed rats even when the diet contained more Kcal could be due to excess activity of the pups. There is controversy on the effect of high carbohydrate consumption on hyperactivity. Prenatal sucrose exposure seems to cause hyperactive behavior in offspring mice and regulates neurobehavioral development. Sucrose intake is considered by some authors as a risk factor for hyperactivity in attention deficit disorder [28,29]. Diets to reduce symptoms of hyperactivity associated with attention deficit disorder include sugar-restricted regimens [30]. In contrast, some papers have concluded that sugar is not a major cause of hyperactivity [31]. 

Our rats showed a decreased consumption of solid food. Food restriction has also been associated with hyperactivity and it is probable that increased physical activity together with food restriction activates brain reward circuits becoming an addictive behavior [32,33,34]. An additional important fact relating hyperactivity and hypertension is that spontaneously hypertensive rats show hyperactivity and have been proposed as a model of hyperactivity in attention deficit disorder [35]. The study of hyperactivity in sucrose fed rats during the critical window might be the aim of another paper.

The effect of mild dehydration due to a lower water intake by rats receiving sucrose on blood pressure regulation could not be determined in the present paper.

### 4.2. Mechanisms Underlying eNOS Expression

eNOS expression was decreased in rats that ingested sucrose during the critical window around weaning and could impact on the increased blood pressure found in this paper. We have previously reported that this change persists until adulthood in rats having an elevated sucrose intake during the critical window [9]. Although the activity of eNOS might also change, we did not explore it in this paper. In this paper we analyzed the three possible peripheral mechanisms underlying eNOS expression at the end of the critical window around weaning (postnatal day 28) that follow:

#### 4.2.1. Possible Role of Insulin in Blood Pressure Regulation 

We studied the possible involvement of insulin on blood pressure regulation after sugar ingestion during the critical window since two different pathways involved in vasoreactivity are activated by this hormone; the PI3K/ PKB/eNOS pathway and the mitogen activated protein kinase (MAPK)/extracellular signal-regulated kinase (ERK) pathway. It has been reported that eNOS is phosphorylated by PKB, increasing NO production causing vasodilation while vasoconstriction is the result of endothelin-1 production as a consequence of the activation of the MAPK pathway [36]. However, insulin levels were not altered in the SP rats and therefore insulin does not seem to have a direct participation in blood pressure regulation. However, the effect of insulin could be due to insulin resistance in endothelial cells [36] which decreases eNOS activity, or to modifications on the downstream substrates of the insulin receptor. 

#### 4.2.2. Fatty Acids and the Expression of eNOS 

Lipidic profile alterations play an important role in insulin resistance and the HOMA-IR and determine vascular reactivity, thus participating in the regulation of elevated blood pressure. The role of triglycerides in hypertension has been previously addressed and an increase in their concentration was found in 28-day old rats receiving sucrose. The elevated levels of triglycerides persist until adulthood in rats that received sucrose during the critical window and then drank tap water for 6 months developing hypertension in adulthood [9]. Elevations in triglycerides have also been reported in metabolic syndrome models in which hypertension is present [37,38]. 

HDL increases phosphorylated eNOS activity. Endothelial cells incubated with HDL show an elevation in the activity of eNOS [38]. HDL cholesterol levels were decreased in rats receiving sucrose during the critical window and therefore could be down regulating eNOS.

HOMA-IR and hypertension are also influenced by high levels of non-esterified FA and of monounsaturated FA [38,39]. Monounsaturated fatty acids were increased in 28 days old rats receiving sucrose. Endothelial cell membrane phospholipid composition is affected by alterations in serum FA and MUFA [40]. The fatty acids participate importantly in regulatory functions, acting as precursors of prostaglandins, thromboxanes and leukotrienes [41]. Changes in endothelial fatty acid composition of the membranes of endothelial cells may diminish access to interaction with receptors, function of membrane ionic transporters and membrane enzymes [42,43]. 

Insulin resistance is related to increased levels of oleic acid. Oleic acid elicits beneficial effects on insulin sensitivity by exerting anti-inflammatory actions, through the reduction of endoplasmic reticulum stress, the attenuation of the insulin signaling pathway, and the improvement of β cell survival rate [44]. Oleic acid (OA) was increased in rats receiving sucrose. Oleic acid causes elevation of ET-1 expression and this response is inhibited by protein kinase C inhibitors and by NF-ĸB inhibitors. In addition, both PKC and NF-κB activities are significantly increased by oleic acid [45]. OA participates in cytokine release, apoptosis, necrosis and oxidative stress [46]. Variations in its levels have been associated to the pathogenesis of endothelial dysfunction and atherosclerosis [47]. OA increases inducible nitric oxide synthase and reduces phosphorylated eNOS expression. Enzymes that control membrane FA biosynthesis such as desaturases have been associated to membrane structure disorders [48,49]. The increase in OA found in rat aortas at the end of the critical window was lost in rats that ingested sucrose during the critical window and were then allowed to drink tap water until adulthood developing hypertension [9]. 

In this paper, stearic and linoleic acids were decreased. Stearic acid is a precursor of oleic acid and its diminution might be caused by the formation of OA. Linoleic acid is a precursor of arachidonic acid. Arachidonic acid also plays a role as a second messenger and modulates intracellular signal transduction [50]. Although arachidonic acid was increased in rats that received sucrose and then tap water until adulthood, there were no important changes in its levels at the end of the critical window. Arachidonic acid elevates iNOS gene expression in cultures of human endothelial cells, [51].

#### 4.2.3. Oxidative Stress and eNOS Expression 

When the balance between the generation of ROS and the antioxidant defenses of the biological system is lost, it results in OS. OS has been reported to participate in the development of cardiovascular diseases such as hypertension, arrhythmias, coronary arterial disease, left ventricular hypertrophy, aortic dilatation, aortic dissection, and congestive heart failure [52,53,54]. OS participates in the regulation of vasoreactivity by altering the contractile function of vascular smooth cells. ROS react with NO diminishing its bioavailability and decreasing endothelium-mediated relaxation [51,55,56]. Moreover, the uncoupled eNOS constitutes a source of ROS. 

One of the effects of the presence of OS is LPO. LPO was increased at the end of the critical window and the total non-enzymatic antioxidant capacity in the aorta was decreased and these conditions might participate as possible causes of hypertension. LPO was not observed in adult hypertensive rats that had received sucrose during the critical window. Reparation of the damages by compensatory mechanisms might have acted during the time these animals drank tap water until adulthood. OS damages tissue and rapid detoxification mechanisms are necessary, as well as processes aimed to repair its damage [51,57]. The total non-enzymatic antioxidant capacity was decreased at the end of the critical window in SP rats. In this paper we found that the expression of one of the anti-oxidant enzymes (SOD2) was decreased at the end of the SP. The reduction in the expression of SOD 2 was also present in adult rats that developed hypertension after having ingested sucrose during the critical window [9]. 

There exists a self-perpetuating cycle between OS and inflammation that participates in the vascular dysfunction and renal damage associated with hypertension. Vascular and renal effects are mediated in part, through the induction of ROS-producing enzymes such as superoxide anion generating nicotinamide adenine dinucleotide phosphate hydrogen (NADPH) oxidases (Nox) and anti-oxidant systems [58,59]. There is increased production of ROS and reactive nitrogen species (RNS) through different pathways such as mitochondrial xanthine oxidase and NADPH oxidase Nox in these pathologies [60,61,62,63,64,65,66]. 

### 4.3. Substrate Availability and Epigenetic Regulated Expression of Hypertension Mediators

Increasing evidence suggests that substrate availability, metabolism and energy production, modulate DNA transcription and epigenetic regulated expression of proteins. Mitochondria play an important role as sensors of the redox and energy state and respond to the chemical environment and levels of intracellular metabolites. Key mitochondrial metabolites such NAD^+^, ATP, α -ketoglutarate and acetyl-CoA are necessary for numerous transcriptional regulations, for protein modifications and for phosphorylation of histones [13,67,68,69,70,71,72]. Therefore, an increase in sucrose during critical windows might be accompanied by the generation of epigenetic marks generated by mitochondrial metabolites, which could modify the expression of enzymes involved in tension development in arteries. 

Substrates abundance increases acetylation of histones. Citrate formed from glucose through the mitochondrial tricarboxylic acid cycle provides the carbon for acetyl-CoA destined for histone acetylation. Acetylation of lysines neutralizes the positive charge of histones decreasing protein affinity for DNA and diminishes protein expression. In contrast, when carbohydrates and fats are reduced, in situations such as fasting or starvation, acetyl-CoA levels are reduced causing reduced acetylation, condensation of chromatin and decreased cellular gene expression, replication and proliferation [13,67,68]. 

Elevated energy supplies result in less oxidation of reduced nicotinamide adenine dinucleotide (NADH) decreasing NAD^+^ levels. The decrease in NAD^+^, inhibits deacetylation of proteins binding to DNA by Sirts. There is a clear crosstalk between Sirt1 and AMPK. Sirt1 and AMPK activate each other and diminish oxidative stress and low-grade inflammation. Conversely, oxidative stress and inflammation diminish AMPK and Sirt1. Sirt1 increases AMP- activated protein kinase (AMPK) phosphorylation and reduces the oxidative damage biomarkers [16]. These factors contribute to insulin resistance and MS–associated disorders which include hypertension [72]. In this paper we studied if the expression of Sirts was modified by a high sucrose diet and if their expression was related to the expression of eNOS and SOD. Sirt1 expression that has been linked to the expression of eNOS [14] was not significantly modified, although there was a tendency for it to increase. In contrast, Sirt3 expression that has been related to SOD2 expression [16] was decreased and this diminution could be related to the reduced expression of the antioxidant enzyme. Changes in SOD2 expression and Sirt3 were also present in adult rats that developed hypertension after having ingested sucrose during the critical window [9]. 

### 4.4. Limitations of the Study

We studied three possible peripheral mechanisms that could participate to increase blood pressure in SP rats; however, mechanisms were not completely clarified and more studies are still needed. Although resistance arteries are more important in regulating blood pressure than conduction vessels such as the aortas, many studies of vascular contractility having relevance on hypertension are done on aortas. Studies of the effect of sucrose during the critical window done in resistance arteries are needed. In this paper, we were unable to determine NO levels which play an important role in blood pressure regulation and its levels should also be measured in the future. The activity of the enzymes studied was not analyzed and could also be involved in the development of hypertension. Histological changes in arteries should also be studied in the future since changes in the structure of the vessel might underlie the functional differences.

## 5. Conclusions

In conclusion, high sugar diets during this critical window increase hypertension predisposition in adulthood and the changes produced by sucrose at the end of the critical window ant the mechanism involves the regulation of eNOS and oxidative stress are important to determine. These changes are present since the end of the critical window and Sirt3 might be underlying the long- lasting effects since it is modified since the end of the critical window. There are long lasting effects determining hypertension susceptibility as a result of changes in the diet during short exposure times in early developmental stages. 

## Figures and Tables

**Table 1:** Body variables and food and water intake in control and sucrose period rats (SP).

Body Variables	Control	Sucrose Period
Body Weight (g)	127.5 ± 1.9	107.6 ± 2.3 ^a^
Visceral Adipose Tissue (mg)	451.3 ± 0.05	461.3 ± 0.06 ^a^
Water intake (mL/day)	34.25 ± 0.98	20.75 ± 0.85 ^a^
kcal equivalents	-	24.9 ± 1.03 ^a^
Food intake (g/day)	15.17 ± 0.96	8.25 ± 0.58 ^a^
kcal equivalents	23.74 ± 1.5	12.91 ± 0.91 ^a^
Total kcal	23.74 ± 1.5	37.81 ± 1.5 ^a^

^a^*p* < 0.05 vs. C, *n* = 6–8 different rat pups from each group.

**Table 2:** Glucose, insulin and insulin resistance in control and sucrose period rats (*n* = 6).

Variables	Control	Sucrose Period
Glucose (mg/dL)	62.41 ± 3.9	58.90 ± 3.3
Insulin (µU/mL)	0.927 ± 02	0.57 ± 01
HOMA-IR	0.185 ± 0.04	0.089 ± 0.02

**Table 3:** Lipidic profile in control and sucrose period rats.

Lipidic Profile	Control	Sucrose Period
Triglycerides (mg/dL)	75.01 ± 4.55	119.73 ± 11.33 ^a^
Total cholesterol (mg/dL)	84.38 ± 4.95	81.49 ± 3.51
C-HDL (mg/dL)	25.31 ± 1.13	18.80 ± 1.62 ^a^
Non-C HDL (mg/dL)	59.07 ± 4.28	62.69 ± 4.50

^a^*p* < 0.05 vs. C, *n* = 6.

**Table 4:** Changes in individual serum fatty acids in control and sucrose period rats.

Fatty Acid (%)	Control	Sucrose Period
Palmitic	27.18 ± 1.42	29.77 ± 0.54
Stearic	13.25 ± 0.55	10.70 ± 0.54 ^a^
Oleic	17.88 ± 2.69	22.93 ± 0.77 ^a^
Linoleic	15.18 ± 0.69	12.19 ± 0.40 ^a^
Arachidonic	8.55 ± 0.70	7.40 ± 0.61

^a^*p* < 0.05 vs. C, *n* = 8.

## Abbreviations

eNOS	endothelial nitric oxide synthase
FA	fatty acids
MS	metabolic syndrome
NO	nitric oxide
OA	oleic acid
OS	oxidative stress
RIA	radioimmunoassay
ROS	reactive oxygen species
SEM	standard errors of the mean
Sirt1-3	sirtuins 1 and 3
SOD	superoxide dismutase
TGs	triglycerides
